# Supplementary material for: Content-rich biological network constructed by mining PubMed abstracts
Source: BMC Bioinformatics. 2004 Oct 8;5:147. doi: 10.1186/1471-2105-5-147 (PMC528731; doi:10.1186/1471-2105-5-147)
Supplement: Additional File 5 — The original Chilibot query results of the term "long-term potentiation (LTP)" and 22 other terms, limiting the latest references analyzed to the years 1990, 1995, 2000, and 2004. [file 1471-2105-5-147-S5.bz2 › chilibotAdditionalFile5/ltp2004/html/ARC_ZIF268.html]

 


 **ARC** and **ZIF268** 
  
Found 8 abstracts in PubMed,  **8 abstracts were retrieved and analyzed**.  


---

 Search Google  |
 PDF files only 
|  EDU domain only 

---

**Interactive relationship** (e.g. stimulation, inhibition, etc)

- **Arc**  RNA expression differed from that of  **zif268**  and c fos in two regards 1 hippocampal  **Arc**  RNA levels were correlated with learning of the hippocampal dependent spatial... water task, and 2  **Arc**  RNA levels in the hippocampus and entorhinal cortex increased after spatial reversal learning relative to an asymptotic performance group.  Ref: 11438584 J Neurosci, 2001

- :-)

  **Parallel relationship** (e.g. studied together, co-existance, homology, etc.)

  - At the age when these animals developed cognitive dysfunction, they had reduced mRNA expression of several genes essential for long term potentiation and memory formation  **Arc** ,  **Zif268** , NR2B, GluR1, Homer 1a, Nur77 TR3.  Ref: 12832546 J Neurosci, 2003
  - Basal  **zif268**  and  **arc**  mRNAs expression was reduced in mutant mice by 60 80%, not only in the striatum but also widespread in the cerebral cortex and hippocampus.  Ref: 11432985 J Neurochem, 2001
  - The time course for the induction of  **zif268**  mRNA and  **Arc**  mRNA by electroconvulsant shock ECS was altered in the area of the dentate gyrus of the hippocampus in rats exposed to lead from postnatal days PND 1 to 28.  Ref: 12111808 J Neurosci Res, 2002
  - When LTP was induced in vitro neither CA1 pyramidal cells nor granule cells showed an increase in  **Zif268** ,  **Arc**  or Homer mRNA.  Ref: 11264669 Eur J Neurosci, 2001
  - Experience dependent gene expression in the rat hippocampus after spatial learning a comparison of the immediate early genes  **Arc** , c fos, and  **zif268** .  Ref: 11438584 J Neurosci, 2001
  - Thus, although the expression of  **Arc** ,  **zif268** , and c fos exhibited many similarities,  **Arc**  was most responsive to differences in behavioral task demands.  Ref: 11438584 J Neurosci, 2001
  - These results establish the existence of subfield specific transcriptional responses to LTP inducing stimulation in the hippocampus of the intact animal, and demonstrate that in area CA1 enhanced transcription of  **Zif268** ,  **Arc**  and Homer is not required for the induction of late LTP.  Ref: 11264669 Eur J Neurosci, 2001
  - As such, localized disruption of IEGs  **Arc**  or c fos by intrahippocampal administration of antisense oligonucleotides or germline disruption of the IEGs c fos, tissue plasminogen activator, or  **zif268**  impairs consolidation of long term memory formation, without affecting learning or short term memory.  Ref: 11918292 Hippocampus, 2002
  - Exposure to lead elevates induction of  **zif268**  and  **Arc**  mRNA in rats after electroconvulsive shock the involvement of protein kinase C.  Ref: 12111808 J Neurosci Res, 2002
  - Because of the importance of gene regulation for neurodevelopment, we examined the effect of lead on the induction of the mRNA of the immediate early genes  **zif268**  and  **Arc** .  Ref: 12111808 J Neurosci Res, 2002
  - The expression of several immediate early genes IEGs  **Arc** , Nur77 and  **Zif268**  and several other plasticity related genes GluR1, CaMKIIalpha and Na K ATPase alphaIII critical for learning and memory was normal in young APP PS1 mice preceding amyloid deposition, but declined as mice grew older and amyloid deposits accumulated.  Ref: 14690531 J Neurochem, 2004
  - By contrast,  **Arc**  and  **zif268**  RNA levels were not correlated or only weakly correlated across structures, although c fos RNA levels were moderately correlated across structures.  Ref: 11438584 J Neurosci, 2001
  - In contrast, we were unable to detect modulation of the IEGs  **Zif268** ,  **Arc** , Homer and HB GAM following induction of LTP by high frequency stimulation of the commissural projection to CA1 pyramidal cells in vivo.  Ref: 11264669 Eur J Neurosci, 2001
  - **Zif268** ,  **Arc**  and Homer can be modulated in CA1 pyramidal cells approximately twofold after electroshock induced maximal seizure, which demonstrates potential responsiveness to electrical stimuli.  Ref: 11264669 Eur J Neurosci, 2001
  - In granule cells of the dentate gyrus, LTP induced in vivo by tetanic stimulation of the perforant path was followed by strong induction of the immediate early genes IEGs  **Zif268** ,  **Arc**  and Homer.  Ref: 11264669 Eur J Neurosci, 2001
  - The objective of the current study was to determine whether the expression of an effector IEG  **Arc**  was similar to, or different from, that of two well characterized RTF IEGs c fos and  **zif268**  after learning.  Ref: 11438584 J Neurosci, 2001
